# Therapeutics and COVID‐19—A living WHO guideline: Endorsement by the Scandinavian Society of Anaesthesiology and Intensive Care Medicine

**DOI:** 10.1111/aas.14046

**Published:** 2022-02-28

**Authors:** Morten H. Møller, Michelle S. Chew, Klaus T. Olkkola, Marius Rehn, Arvi Yli‐Hankala, Martin I. Sigurðsson

**Affiliations:** ^1^ Department of Intensive Care Copenhagen University Hospital Rigshospitalet Copenhagen Denmark; ^2^ Department of Anaesthesia and Intensive Care Biomedical and Clinical Sciences Linköping University Linköping Sweden; ^3^ Department of Anaesthesiology, Intensive Care and Pain Medicine University of Helsinki and Helsinki University Hospital Helsinki Finland; ^4^ Division of Prehospital Services Air Ambulance Department Oslo University Hospital Oslo Norway; ^5^ The Norwegian Air Ambulance Foundation Drøbak Norway; ^6^ Faculty of Health Sciences University of Stavanger Stavanger Norway; ^7^ Department of Anaesthesia Tampere University Hospital Tampere Finland; ^8^ Faculty of Medicine and Health Technology Tampere University Tampere Finland; ^9^ Division of Anesthesia and Intensive Care Medicine Landspitali‐The National University Hospital of Iceland Reykjavik Iceland; ^10^ Faculty of Medicine University of Iceland Reykjavik Iceland

**Keywords:** AGREE II, clinical practice guideline, COVID‐19, therapeutics, WHO

## Abstract

The Clinical Practice Committee of the Scandinavian Society of Anaesthesiology and Intensive Care Medicine endorses the *Living WHO guideline on therapeutics and COVID*‐*19*. This trustworthy continuously updated guideline serves as a highly useful decision aid for Nordic anaesthesiologists caring for patients with COVID‐19.

## 1 BACKGROUND

Severe acute respiratory syndrome coronavirus‐2 (SARS‐CoV‐2) has caused a pandemic of coronavirus disease 2019 (COVID‐19),^1^ including in the Nordic countries.^2^ Many patients have suffered from severe hypoxic respiratory failure and have died, and most healthcare systems worldwide have been overwhelmed by the surge of patients needing hospitalisation and intensive care.^3^, ^4^

The *World Health Organization (WHO) living guideline on therapeutics in COVID*‐*19* continuously summarises the emerging evidence from randomised controlled trials on existing and new drug treatments for COVID‐19.^5^

## 2 METHODS

It was decided by the Clinical practice committee (CPC) of the Scandinavian Society of Anaesthesiology and Intensive Care Medicine (SSAI) to assess the 7th *edition of the Living WHO guideline on therapeutics and COVID*‐*19* ^5^ for possible endorsement. The Appraisal of Guidelines for REsearch and Evaluation (AGREE) II tool^6^ was used. Details on the endorsement process are available elsewhere.^7^

## 3 RESULTS

**FIGURE 1:**
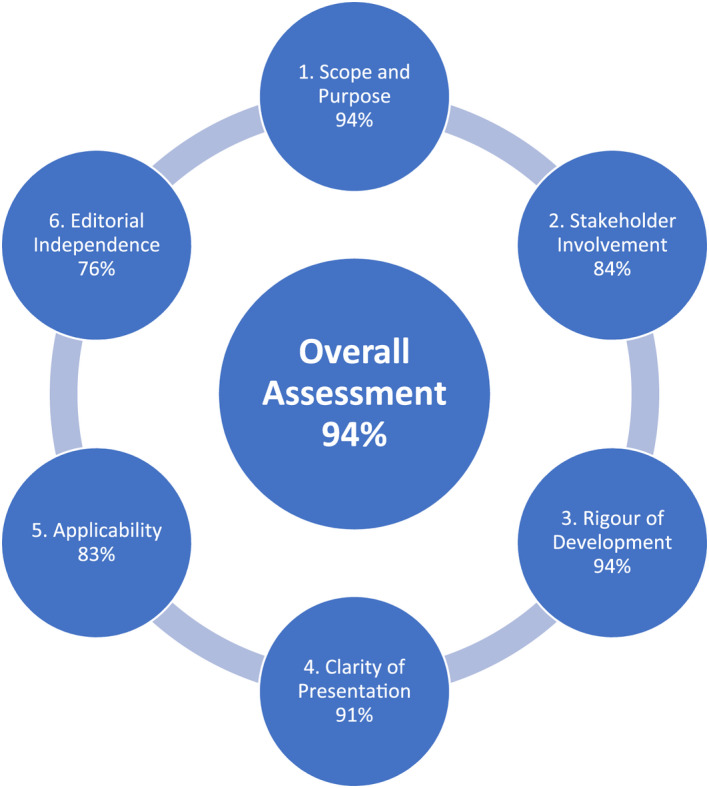
Summary of the Appraisal of Guidelines for REsearch and Evaluation (AGREE) II assessment^6^

All six SSAI CPC members completed the appraisal. The individual domain totals were: Scope and Purpose 94%; Stakeholder Involvement 84%; Rigour of Development 94%; Clarity of Presentation 91%; Applicability 83%; Editorial Independence 76%; Overall Assessment 94% (Figure 1).

The breakdown of the individual appraisers (de‐identified) is available in the Supporting information.

## 4 DISCUSSION

Agreement between the SSAI CPC appraisers was high and the overall assessment of the guideline was very good. The guideline can be used in daily clinical practice in Nordic countries without adaptation or modification. Of note, the effects of the proposed therapeutic agents may to some extent be sensitive to the specific settings they are used in, including the baseline disease severity of COVID‐19.

The *Living WHO guideline on therapeutics and COVID*‐*19* ^5^ serves as a highly useful decision aid for Nordic anaesthesiologists caring for patients with COVID‐19. Detailed recommendations, evidence summaries, and decision aids are available in MAGICapp (https://app.magicapp.org/#/guideline/nBkO1E).

## 5 CONCLUSION

The SSAI CPC endorses the *Living WHO guideline on therapeutics and COVID*‐*19*.^5^

## CONFLICTS OF INTEREST

No Clinical Practice Committee member had direct or indirect conflicts of interest.

## Supporting information

Supplementary Material
